# How to Predict Molecular Interactions between Species?

**DOI:** 10.3389/fmicb.2016.00442

**Published:** 2016-03-31

**Authors:** Sylvie Schulze, Jana Schleicher, Reinhard Guthke, Jörg Linde

**Affiliations:** Research Group Systems Biology and Bioinformatics, Leibniz-Institute for Natural Product Research and Infection Biology – Hans-Knöll-InstituteJena, Germany

**Keywords:** dual transcriptomics, dual RNA-seq, gene regulatory network, molecular inter-species interaction, network inference, host-pathogen interaction

## Abstract

Organisms constantly interact with other species through physical contact which leads to changes on the molecular level, for example the transcriptome. These changes can be monitored for all genes, with the help of high-throughput experiments such as RNA-seq or microarrays. The adaptation of the gene expression to environmental changes within cells is mediated through complex gene regulatory networks. Often, our knowledge of these networks is incomplete. Network inference predicts gene regulatory interactions based on transcriptome data. An emerging application of high-throughput transcriptome studies are dual transcriptomics experiments. Here, the transcriptome of two or more interacting species is measured simultaneously. Based on a dual RNA-seq data set of murine dendritic cells infected with the fungal pathogen *Candida albicans*, the software tool NetGenerator was applied to predict an inter-species gene regulatory network. To promote further investigations of molecular inter-species interactions, we recently discussed dual RNA-seq experiments for host-pathogen interactions and extended the applied tool NetGenerator (Schulze et al., 2015). The updated version of NetGenerator makes use of measurement variances in the algorithmic procedure and accepts gene expression time series data with missing values. Additionally, we tested multiple modeling scenarios regarding the stimuli functions of the gene regulatory network. Here, we summarize the work by Schulze et al. (2015) and put it into a broader context. We review various studies making use of the dual transcriptomics approach to investigate the molecular basis of interacting species. Besides the application to host-pathogen interactions, dual transcriptomics data are also utilized to study mutualistic and commensalistic interactions. Furthermore, we give a short introduction into additional approaches for the prediction of gene regulatory networks and discuss their application to dual transcriptomics data. We conclude that the application of network inference on dual-transcriptomics data is a promising approach to predict molecular inter-species interactions.

## Introduction

**Figure 1:**
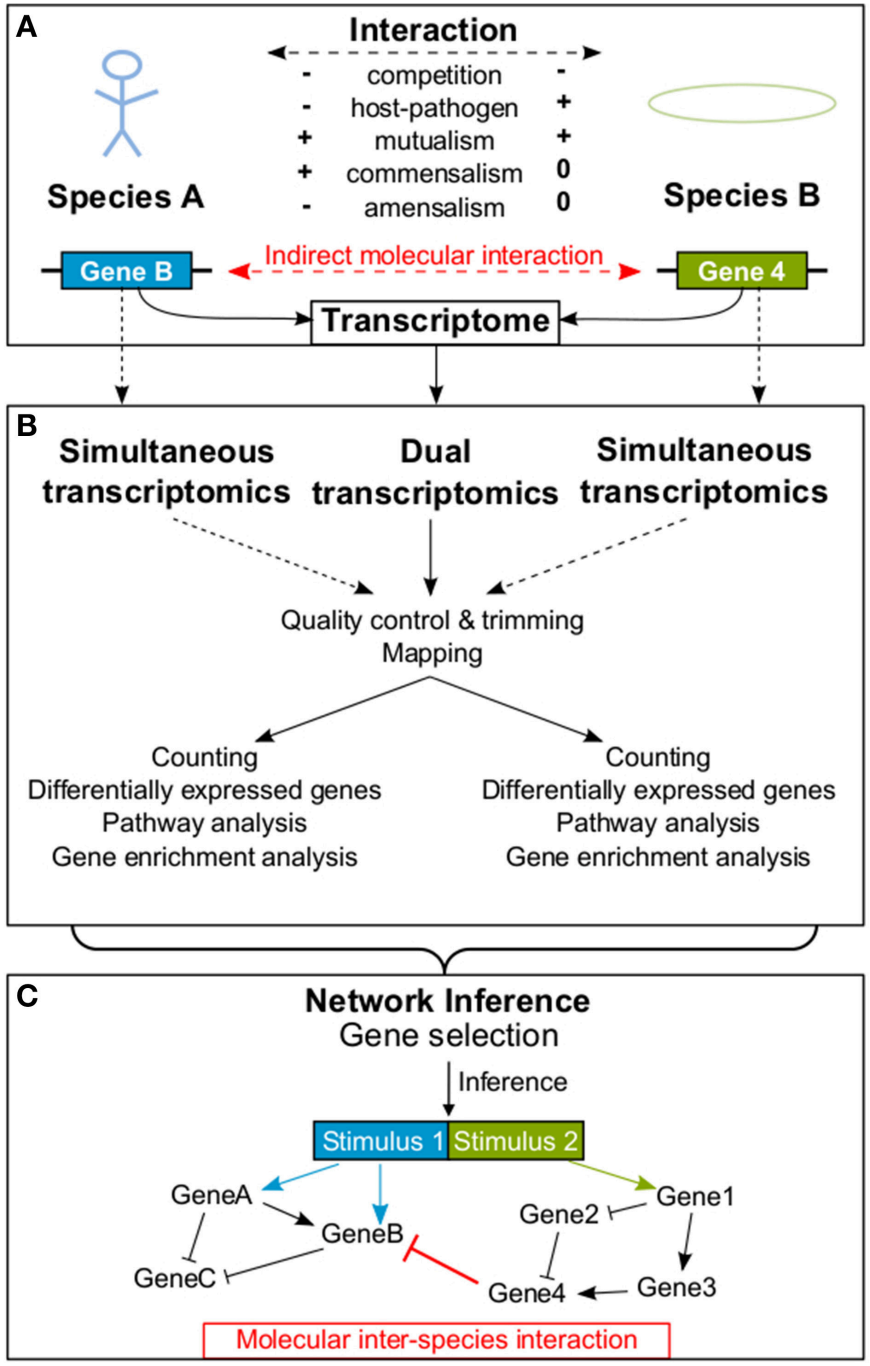
**An example is provided how a molecular interaction between two species is detected**. Panel **(A)** provides a schematic overview of possible interactions between organisms of two different species influencing each other's transcriptome. In **(B)** the processing of dual RNA-seq data extracted simultaneously from both interacting species is shown. In contrast, dotted lines represent simultaneous transcriptomics, where both transcriptomes are analyzed separately. In **(C)** an exemplary GRN resulting from the inference process is illustrated, including an indirect molecular inter-species interaction between two genes (red bar). In addition, molecular intra-species interactions (black) within each of the two species are shown. Arrowheads indicate activation and bars indicate repression.

Organisms constantly interact with their abiotic and biotic environment (Koshland, 2002). Generally, biotic interactions are characterized by their effects on the fitness of an organism (Figure 1A): The result of an interaction can be a fitness gain (+), a fitness loss (−), or has no effect on fitness (0). Based on the interaction outcome for the organisms fitness, researchers can classify biotic interactions into competition (−∕−), predator-prey interaction (+∕−), parasite/pathogen-host interaction (+∕−), mutualism (+∕+; including symbiosis), commensalism (+∕0), and amensalism (−∕0) (Begon et al., 2006).

Most of these interactions do not only appear on the macroscopic (e.g., organism interaction) or mesoscopic level (e.g., cell-cell interaction), but also affect the molecular level of the cells through molecular mediators. Thus, interactions can be scaled down to changes in gene expression, i.e., to the transcriptome. Biotic interactions change the organisms' environment. These changes, but also the interaction partner themselves, are sensed by receptors on cell surfaces and transmitted into the cell by signaling cascades. Such signals finally (in)activate transcriptional regulators (Groisman and Mouslim, 2006). These regulators alter the expression of their target genes which may in turn regulate other target genes within complex **Gene Regulatory Networks (GRNs)** (Barabási and Oltvai, 2004; Emmert-Streib et al., 2014). These specific and complex networks determine the cells response to changes caused by a macroscopic interaction. Currently, our knowledge about these complex GRNs is limited, but it would be of great help to understand the molecular basis of biotic interactions taking place on the macroscopic level.

KEY CONCEPT 1 **Gene regulatory networks:**

A GRN consists of regulatory interactions between genes in order to adjust the mRNA expression levels to an applied stimulus. Commonly, they are visualized as nodes representing genes and edges representing regulatory interactions.

Transcriptomics offers a comprehensive way to study expression changes of all genes of an organism under different conditions (e.g., reviewed by Jenner and Young, 2005; Kammenga et al., 2007; Leroy and Raoult, 2010). Traditionally, microarrays have been applied for transcriptomics. Since the advent of next-generation-sequencing of cDNAs derived from RNA samples (**RNA-seq**), researchers are able to study transcriptomes with a higher sensitivity and unlimited detection ranges (Mardis, 2008; Wang et al., 2009). One advantage of RNA-seq over microarrays is that RNA-seq offers a species-independent platform which allows for investigations of non-model species.

KEY CONCEPT 2 **RNA-seq:**

RNA-seq is a next-generation-sequencing technology where gene expression levels are measured based on high-troughput sequencing of RNA molecules.

Given that an interaction between organisms of two different species affects each one's gene expression, it is promising to study the transcriptomes of both species simultaneously in order to understand the molecular basis of the observed interaction. **Dual transcriptomics** is characterized by simultaneous measurements of the transcriptome from two interacting species where the processing of samples occurs collectively and species specific expression is determined *in silico* (Westermann et al., 2012). Dual transcriptomics has been successfully applied using both microarrays and RNA-seq and contributed to new knowledge about important biological questions (see Section 2; Table 1). For example, dual transcriptomics data allows to determination of which genes or proteins indirectly or directly interact between two species. Of note, this approach is different to metagenomics, which identifies the entire genome representation of an (environmental) sample; and it is different to comparative transcriptomics, which compares the responses of different species to the same stimulus. Strictly speaking, dual transcriptomics is not the same as simultaneous transcriptomics, where the transcriptome of two interacting species is captured simultaneously but processing is carried out separately for each species/RNA sample (e.g., Oosthuizen et al., 2011; Vojvodic et al., 2015). In what follows, we differentiate between simultaneous and dual transcriptomics, but proposed approaches may work for both.

KEY CONCEPT 3 **Dual transcriptomics:**

Dual transcriptomics means to simultaneously measure the transcriptome of two interacting species and collectively preprocess samples. Species specific gene expression is determined *in silico*. Typically, RNA-seq or microarrays are utilized for this purpose.

**Table 1:** **Selection of published studies investigating molecular interactions between organisms by applying simultaneous and dual transcriptomics approaches**.

**Interaction modus**	**References**	**Interacting species**	**Method**
**HOST-PATHOGEN INTERACTION**			
Parasite-plant	(Moy et al., 2004) Patterns of gene expression upon infection of soybean plants by *Phytophthora sojae*.	Oomycete *Phytophthora sojae* - Soybean *Glycine max*	Dual microarray
Parasite-plant	(Ithal et al., 2007) Parallel genome-wide expression profiling of host and pathogen during Soybean cyst nematode infection of soybean.	Nematode *Heterodera glycin* - Soybean *Glycine max*	Dual microarray
Pathogen-plant	(Hayden et al., 2014) Dual RNA-seq of the plant pathogen *Phytophthora ramorum* and its tanoak host.	Fungus-like *Phytophthora ramorum* - Tree *Notholithocarpus densiflorus*	Dual RNA-seq
Fungus-plant	(Eaton et al., 2010) Exploring molecular signaling in plant-fungal symbioses using high throughput RNA sequencing.	Fungus *Epichloë festucae* - Legume *Lolium perenne*	Simultaneous RNA-seq
Fungus-plant	(Asai et al., 2014) Expression profiling during arabidopsis/downy mildew interaction reveals a highly-expressed effector that attenuates responses to salicylic acid.	Fungus *Hyaloperonospora arabidopsidis* - Plant *Arabidopsis thaliana*	Dual RNA-seq
Fungus-plant	(Teixeira et al., 2014) High-resolution transcript profiling of the atypical biotrophic interaction between *Theobroma cacao* and the fungal pathogen *Moniliophthora perniciosa*.	Fungus *Moniliophthora perniciosa* - Plant *Theobroma cacao*	Dual RNA-seq
Fungus-plant	(Lowe et al., 2014) Genomes and transcriptomes of partners in plant-fungal interactions between canola (*Brassica napus*) and two *Leptosphaeria* species.	Fungus *Leptosphaeria* species - Plant *Brassica napus*	Dual RNA-seq
Fungus-human	(Tierney et al., 2012) An interspecies regulatory network inferred from simultaneous RNA-seq of *Candida albicans* invading innate immune cells.	Fungus *Candida albicans* - *Mus musculus* (dentritic cells)	Dual RNA-seq
Fungus-human	(Liu et al., 2015) New signaling pathways govern the host response to *C. albicans* infection in various niches.	Fungus *Candida albicans* - Human endothelial cells/oral epithelial cells (*in vitro* infection)	Dual RNA-seq
Fungus-human	(Oosthuizen et al., 2011) Dual organism transcriptomics of airway epithelial cells interacting with conidia of *Aspergillus fumigatus*.	Fungus *Aspergillus fumigatus* - Human airway epithelial cells	Simultaneous microarray
Bacterium-human	(Humphrys et al., 2013) Simultaneous transcriptional profiling of bacteria and their host cells.	Bacterium *Chlamydia trachomatis* - Human epithelial cell *in vitro*	Dual RNA-seq
Parasite-human	(Yamagishi et al., 2014) Interactive transcriptome analysis of malaria patients and infecting *Plasmodium falciparum*.	Parasite *Plasmodium falciparum* - Human (malaria patients)	Dual RNA-seq
Fungus-animal	(Bruno et al., 2015) Transcriptomic analysis of vulvovaginal candidiasis identifies a role for the NLRP3 inflammasome.	Fungus *Candida albicans* - Mouse (*Mus musculus*)	Dual RNA-seq
Virus-animal	(Rosani et al., 2014) Dual analysis of host and pathogen transcriptomes in ostreid herpesvirus 1-positive *Crassostrea gigas*.	Ostreid herpesvirus type 1 - pacific oyster *Crassostrea gigas*	Dual RNA-seq
Parasite-animal	(Foth et al., 2014) Whipworm genome and dual-species transcriptome analyses provide molecular insights into an intimate host-parasite interaction.	Nematode *Trichuris spec*. - Mouse *Mus musculus*	Dual RNA-seq
**INTERACTION MODUS**			
Bacterium-animal	(Brogaard et al., 2015) Concurrent host-pathogen gene expression in the lungs of pigs challenged with *Actinobacillus pleuropneumoniae*.	Bacterium *Actinobacillus pleuropneumoniae* - Pig	High-throughput RT-qPCR
Nematode-insect	(Choi et al., 2014) Dual RNA-seq of parasite and host reveals gene expression dynamics during filarial worm-mosquito interactions.	Nematode *Brugia malayi* - Mosquito *Aedes aegypti*	Dual RNA-seq
**HOST-SYMBIONT INTERACTION**			
Bacterium-plant	(Roux et al., 2014) An integrated analysis of plant and bacterial gene expression in symbiotic root nodules using laser-capture microdissection coupled to RNA sequencing.	Bacterium *Sinorhizobium meliloti* - Legume *Medicago truncatula*	Dual RNA-seq
Fungus-plant	(Handa et al., 2015) RNA-seq transcriptional profiling of an arbuscular mycorrhiza provides insights into regulated and coordinated gene expression in *Lotus japonicus* and *Rhizophagus irregularis*.	Fungus *Rhizophagus irregularis* - Legume *Lotus japonicus*	Dual RNA-seq
Fungus-plant	(Johnson et al., 2007) Dual Affymetrix GeneChip(R) analysis of the perennial ryegrass-endophyte symbiosis.	Fungus *Epichloë festucae*/Fungus *Neotyphodium lolii* - Legume *Lolium perenne*	Dual microarray
**SOCIAL INSECT INTERACTION**			
	(Vojvodic et al., 2015) The transcriptomic and evolutionary signature of social interactions regulating honey bee caste development.	Developing honey bee larvae - Caregiving adult worker	Simultaneous RNA-seq
**SYMBIONT-SYMBIONT INTERACTION**			
Bacterium-bacterium	(Rosenthal et al., 2011) RNA-seq reveals cooperative metabolic interactions between two termite-gut spirochete species in co-culture.	Bacterium *Treponema primitia* - Bacterium *Treponema azotonutricium*	Dual RNA-seq

Using dual transcriptomics, researchers have identified important genes, pathways and processes during the interaction of interacting species. However, so far only a few studies have elucidated more specifically which gene or pathway in organism A affects which gene or pathway in organism B (Table 1). Moreover, if such information is given it is either based on previous knowledge or was found by small scale experiments. In most studies, underlying transcriptome data were not used to infer molecular interactions between species.

**Network Inference (NI)** is a Systems Biology approach that predicts molecular regulatory interactions between genes in GRNs from gene expression data. There are different methodological approaches which were successfully applied in a number of studies (reviewed in Hecker et al., 2009b; Emmert-Streib et al., 2014; Linde et al., 2015). Commonly, NI has been applied to predict a GRN in one species. In a pioneering study, we applied NI on **dual RNA-seq** data of the fungal pathogen *Candida albicans* interacting with dendritic cells of *Mus musculus*. We predicted two molecular host-pathogen interactions, which were experimentally validated with help of intracellular staining, cellular binding assays as well as knock-outs of fungal genes and knock-downs of mouse genes followed by rtPCRs (Tierney et al., 2012). To our best knowledge, this is currently the only study which applied NI on dual-transcriptomics data. Guided by this experience, we augmented our NI approach to deal with typical situations for NI based on dual transcriptomics data. In Schulze et al. (2015), we published an improved version of our NI tool NetGenerator (Guthke et al., 2005; Toepfer et al., 2007) —an algorithm to infer GRNs based on time series gene expression data that are simulated by Ordinary Differential Equations (ODEs) and includes prior knowledge (see Section 4). NetGenerator is now capable of dealing with missing data and has more options for handling variance of gene expression data. In addition, we tested the influence of integrating multiple stimuli.

KEY CONCEPT 4 **Network inference:**

Network inference is a modeling approach to predict gene regulatory networks based on gene expression data.

KEY CONCEPT 5 **Dual RNA-seq:**

The term dual RNA-seq describes the simultaneous sequencing of mixed RNA-pools originating from two (interacting) species. When a pool of sequenced reads is mapped to genomes, reads are separated and mapped to the genome of the species they originate from.

This Frontiers Focused Review puts the results of Schulze et al. (2015) into a broader context. In addition to dual transcriptome studies dealing with host-pathogen interactions, we review studies from other fields of biology, such as mutualistic interactions. Furthermore, we give an overview of other NI approaches and discuss their possible applications to dual transcriptomics. Finally, we outline the usability of novel extensions proposed in Schulze et al. (2015) and discuss current problems as well as future developments.

## Dual transcriptomics

The term dual RNA-seq describes the transcriptome sequencing of two or more interacting species based on one mixed RNA-pool. This approach of measuring the transcriptome of two different species in one run has multiple advantages over simultaneous transcriptomics. RNA can be extracted directly without separating interacting species. Therewith, measured gene expression data directly reflects changes due to macroscopic interactions and not side effects of separation. Also, sequencing costs are lower because only one library is constructed. In some experimental setups interacting species are separated, their transcriptomes are sequenced in two runs and data processing is carried out separately. We refer to this procedure as simultaneous transcriptomics.

Raw dual RNA-seq data are preprocessed as follows (Figure 1B): Low quality bases of reads are trimmed [e.g., Trimmomatic (Bolger et al., 2014), CutAdapt (Martin, 2011)] and samples are quality controlled [e.g., HTQC (Yang et al., 2013), FastQC (Andrews, 2010)]. When the quality report is checked, researchers need to keep species properties in mind. E.g., the per sequence GC content plot of the FastQC report could show two peaks, if the GC content of investigated species is very different. A crucial step in dual RNA-seq preprocessing is the alignment of reads to their corresponding genomes (“mapping”; reviewed in Engström et al., 2013; Shang et al., 2014). Reads can be mapped consecutively to both genomes, but the order will influence the results since some reads may map to both genomes (depending on their evolutionary distance, sequencing parameters and read length). Alternatively, reads can be mapped in parallel to concatenated genomes. The advantage is, that in case of possible alignments to both genomes, the read mapping tools find the best position. The drawback is, that in case of equally good alignments to both genomes the read will be discarded as multi-mapped read. Also, mapping parameters are crucial, e.g., if intron lengths of studied organisms are very different, a tool non-sensitive to this parameter should be applied. In advance to carrying out experiments, RNA ratios of studied organisms should be determined and reflected by the mapping rates. As a next step, expression values of features (e.g., genes or transcripts) are calculated [e.g., featureCounts (Liao et al., 2014), maxcounts (Finotello et al., 2014)] and testing for differential expression is carried out separately for each species (reviewed in Rapaport et al., 2013; Soneson and Delorenzi, 2013; Zhang et al., 2014).

Sequencing parameters, such as rRNA filtering (filter RNA species of interest) (Westermann et al., 2012), read length (number of sequenced bases of a RNA fragment), single- vs. paired-end libraries (sequence a fragment from one or two ends), sequencing depth (average number how often every base is sequenced), and number of replicates (biological or technical), need to be chosen carefully depending on the species of interest and project aims. For example, the extractable RNA amounts of each species need to be determined and optimized in advance to the sequencing experiment. Therewith, a minimal sequencing depth to achieve sufficient coverage for all species can be calculated. If the species of interest are closely related, researchers might sequence longer paired-end reads to better determine read-genome correspondence and to prevent a large proportion of multi-mapped reads. If researchers aim for a highly reproducible detection of Differentially Expressed Genes (DEGs), Liu et al. (2013) recommended to generate more biological replicates rather than a higher sequencing depth. Nevertheless, if the aim is to detect DEGs with low expression, a high sequencing depth is necessary. Generally, we recommend to generate at least three replicates with a minimum coverage of ~10 fold to detect DEGs.

In dual or simultaneous transcriptome studies based on microarrays, RNA is extracted separately from each species and hybridized to species-specific microarrays. Therefore, preprocessing is not different from standard microarray data preprocessing. Even though, microarray data preprocessing slightly differs for different technological platforms (Gautier et al., 2004; Du et al., 2008), the main steps are (Irizarry et al., 2003): (i) image processing including background substraction, (ii) within array normalization to correct for spatial effects or cross-hybridization on each array, (iii) between array normalization to ensure that expression values have the same empirical distribution across different arrays/slides and (iv) testing for DEGs between conditions. Here, empirical t-statistics combined with a multiple test correction and a fold-change cutoff have emerged as a standard (Smyth, 2005; SEQC/MAQC-III Consortium, 2014).

## Dual transcriptomics applications

Over the last years, the use of simultaneous transcriptomics to elucidate the molecular interactions between organisms of the same or of different species has gained increasing importance. One major application of simultaneous transcriptomics is the research area of infectious diseases. Different approaches dealing with the simultaneous analysis of expression profiles from two different species, *via* microarrays or RNA-seq, were published (e.g., Motley et al., 2004; Tierney et al., 2012; Humphrys et al., 2013; Schulze et al., 2015). These methods provide the basis to reveal the complex interplay between invading pathogens and their host. In the following, we briefly review recent simultaneous transcriptomics studies focusing on host-pathogen interactions. In addition, we provide insights to other relevant fields of biological interactions in which simultaneous transcriptomics plays an increasing role (Table 1).

Host-pathogen interactions are relevant in plant ecology due to their consequences for agricultural ecosystems. Eaton et al. (2010) performed high throughput sequencing of both, a plant host and its pathogen to evaluate interactions in a grass-fungal system. The most important fungal genes responsible for the shift of the fungus from a symbiont to a pathogen were revealed. The study data indicates that the protein sakA is important for the switch from a symbiotic to a pathogenic interaction.

On the plant side, changes in the hormone balance and upregulation of the defense response (generally absent in a symbiotic association) were observed to take place during the change of interaction modes. Interestingly, the same grass-fungal system has been investigated in regard to the symbiotic interaction based on a specifically designed dual microarray, which contains probes of two species (Johnson et al., 2007). In both publications, the symbiotic interaction of the fungus *Epichloë festucae* and the host *Lolium perenne* is investigated which is characterized by the fungal biosynthesis of secondary metabolites that protect the plant from various biotic and abiotic stresses, while the plant provides nutrients to the fungus and a mechanism of dissemination via seed transmission.

Moy et al. (2004) designed a dual microarray to simultaneously measure gene expression of soybean (*Glycine max*) and its pathogen *Phytophthora sojae* on a single array. The authors identified plant genes which are up- or downregulated within 24 h after infection. Analyzing these gene sets, they conclude that during the infection process the pathogen changes from biotrophy to necrotrophy. Similar work in the field of plant-pathogen interaction has been done by Ithal et al. (2007), who investigated the gene expression changes in soybean (*G. max*) and the soybean cyst nematode based on a dual microarray expression study during the course of infection. In addition, Teixeira et al. (2014) used dual RNA-seq to simultaneously assess the transcriptomes of cacao (*Theobroma cacao*) and the fungal pathogen *Moniliophthora perniciosa*, which causes Witches broom disease in its host. The authors found that the pathogen causes a change of the host metabolism to increase nutrition availability. Accordingly, they observed carbon deprivation on the host side and showed that the fungus causes massive metabolic reprogramming in infected shoots.

The fungus *C. albicans* attracts broad research interest due to its ability to switch from a commensal organism to a pathogen which can cause fatal invasive infections in humans (Cheng et al., 2012). To understand the mechanisms involved in the infection process, predominantly one-sided expression analyses focusing on either the host (e.g., Barker et al., 2005; Kim et al., 2005; Fradin et al., 2007) or the pathogen (e.g., Fradin et al., 2003; Fernández-Arenas et al., 2007; Bruno et al., 2010) were conducted in the past. In recent years, insights in the infection and defense mechanisms were gained by simultaneously measuring the hosts and the fungus gene expression profiles utilizing dual RNA-seq (Tierney et al., 2012; Bruno et al., 2015). In the latest study, Liu et al. (2015) identified several active pathways during *C. albicans* and host endothelial cell interaction based on dual RNA-seq data. Furthermore, they validated that two of these pathways regulate the uptake of *C. albicans* by host cells.

Dual transcriptomics was also applied to study host-virus and host-parasite interactions. Dual RNA-seq analysis of pacific oyster (*Crassostrea gigas*) infected by the ostreid herpesvirus type 1 allowed the exploration of the virus transcriptome and the host innate immune response during the process of infection (Rosani et al., 2014, see also Segarra et al., 2014). Furthermore, to understand the molecular mechanisms of parasitism, a dual RNA-seq approach was used to elucidate the transcriptome of malaria patients and the parasite *Plasmodium falciparum* based on blood samples (Yamagishi et al., 2014). The authors found characteristic expression changes of human innate immune response pathways involving TLR2 and TICAM2. Moreover, these expression changes correlated with the severity of the malaria infection.

Besides promoting our understanding of host-pathogen interactions, some studies used dual transcriptomics to investigate mutualism between species. Mycorrhiza is the symbiotic association of certain fungal species with plant roots (Brundrett, 2009). Handa et al. (2015) analyzed the gene expression profiles of the legume *Lotus japonicus* and the mycorrhizal fungus *Rhizophagus irregularis* during root mycorrhizal development. Some highly co-regulated transcripts encoding membrane traffic-related proteins, transporters and iron transport-related proteins were identified. An expression change of fungal cytochrome P450 was measured and the authors hypothesize, that this might contribute to metabolic pathways required to accommodate roots and soil.

Furthermore, an interesting study of mutualism has been conducted by Rosenthal et al. (2011), who investigated the interplay between two termite gut symbionts (spirochetes) using dual RNA-seq. The authors identified detailed cooperative interaction concerning metabolism during interaction of the symbionts. Another application field is the use of dual RNA-seq in plant-bacteria symbiotic interactions. For example, Roux et al. (2014) applied dual RNA-seq on the model legume *Medicago truncatula* and its symbiont *Sinorhizobium meliloti*. Since their expression study was coupled to laser microdissection of nodule regions, authors were able to analyze region sepcific gene expression. The authors found that bacterial transcription factors which control the root apical meristem are also expressed in the nodule meristem. In contrast, plant genes which are higher expressed in nodules than in roots are often associated with regions comprising both plant and bacterial partners.

Recently, simultaneous RNA-seq analysis was applied to elucidate the molecular interaction of social insects. The interplay between developing female honey bee larvae and adult nurse workers was analyzed on the molecular level before and after removal of the queen. By comparing these gene expression profiles, larval and nurse genes associated with caste development were identified (Vojvodic et al., 2015).

This short overview of interaction studies highlights the far-reaching applicability and practicability of dual RNA-seq analysis in the field of biological interactions. Consequently, further development and improvement of suitable methods and approaches, as the prediction of molecular interactions (Schulze et al., 2015), is essential to promote our understanding of the interplay between organisms on the molecular level. This is not only crucial in the research area of infectious diseases, but may also open up research on many symbiotic systems (Eaton et al., 2011) and other biological relevant interaction systems.

## Network inference

**Network Inference** approaches predict GRNs based on gene expression data. The structure of a GRN is thereby reconstructed from the gene expression data in response to an applied stimulus (reverse engineering).

The input for NI approaches is a gene expression matrix, which contains the expression values (or changes) of genes during treatment with different stimuli and/or over time. The number of included genes depends on the research question and on the applied NI approach. Importantly, the number of possible network structures increases exponentially with the number of included genes (curse of dimensionality). Several strategies are used to overcome the curse of dimensionality. Guided by the observation that biological networks have fewer interactions than expected in random networks, many NI approaches apply the sparseness criterion, i.e., predict the smallest number of interactions needed to fit the measured data. Another property of GRNs which is often applied as a network selection criterion is scale-freeness. This means that the number of interaction partners per gene is power law distributed, i.e., most genes interact with a very low number of genes while a few genes (hubs) have a high number of interaction partners (Barabási and Oltvai, 2004). So called prior knowledge are interactions extracted from literature or additional data sources, such as the occurrence of transcription factor binding sites in promoters. Integration of prior knowledge during NI strongly improves the accuracy of predicted interactions (Hecker et al., 2009a).

The result of a NI can be a correlation network, a Bayesian network or a mathematical model, depending on the underlying NI approach. NI approaches differ in the details of predicted interactions. They may predict either undirected interactions (A and B interact) or directed interactions (A regulates B). Directed interactions may additionally be signed (A induces B, A represses C). It is possible to get a steady or a dynamic network model depending on the NI approach. In dynamic network models, the state at a certain time point depends on its state at previous time points. Dynamic network models can be applied to predict future behavior of a system.

The assessment of NI approaches is difficult as they are often applied to very different research questions and success in one experimental setup does not guarantee success in another one. Since 2006, the “Dialog for Reverse Engineering Assessment and Methodsâ” (DREAM, www.dreamchallenges.org) has launched annual competitions for systems biology methods including NI (Stolovitzky et al., 2007).

In what follows, we give a brief and general description of the most important NI approaches and discuss their advantages and disadvantages. We guide the interested reader to excellent reviews for more comprehensive and detailed overviews of NI approaches (Hecker et al., 2009b; Wu and Chan, 2012; Emmert-Streib et al., 2014; Linde et al., 2015).

### Approaches based on correlation and information theory

One of the most straightforward methods to predict a GRN is to compute the pairwise correlation between each pair of genes. An interaction is predicted if the correlation value is above a user-defined cut-off. This approach is computationally very fast and can be applied to a large number of genes. As the concept of correlation is well-known, results are easy to interpret. However, correlation does not mean causality. For example, consider a transcription factor inducing two target genes. The target genes have a high correlation value but do not interact.

To overcome this problem, approaches based on information theory compute the mutual information based on the pairwise correlation matrix. This term measures the statistical dependency between two random variables, which represent the expression intensities of two genes. Several mutual information based approaches are available (Butte and Kohane, 2000; Basso et al., 2005; Faith et al., 2007; Meyer et al., 2007; Altay and Emmert-Streib, 2010). Typically, these approaches do not integrate prior knowledge, nor do they enforce sparseness or scale-freeness. In general, they infer static undirected networks, but augmentations to generate directed networks exist (Madar et al., 2010).

### Bayesian networks

Another probabilistic approach is Bayesian NI. Here, the expression of each gene is considered to be a random variable which follows a probability distribution. Applying the Bayesian theorem, algorithms sample networks from a prior distribution and the network which best explains the measured data is selected. With help of the prior distribution of networks, prior knowledge can be elegantly integrated. Inferred GRNs are directed and can be static or dynamic (without direct feedback). Bayesian NI approaches (Murphy and Mian, 1999; Hartemink et al., 2001; Rau et al., 2010; Yeung et al., 2011) do not directly apply scale-freeness but this criterion might be included in the prior distribution. A major disadvantage is that accuracy of predicted interactions strongly depends on a relatively high amount of measured expression data.

### Linear regression based NI

By applying linear regression, the expression of a gene at condition (time point) t is modeled as the weighted sum of the expression of all other genes. Additionally, some approaches include an external stimulus as part of the weighted sum. This represents the change in the environment. The values for the weights are determined by optimization algorithms in order to fit to the measured expression data. Non-zero weights define the network structure, where a positive weight represents an activation and a negative weight a repression. Thus, linear regression results in directed and signed steady networks. Many linear regression approaches apply the sparseness criterion. They predict the GRN which has a minimal (or small) number of interactions but is still able to fit the measured data. Moreover, they softly integrate prior knowledge (e.g., Gardner et al., 2003; Toepfer et al., 2007; Zou and Hastie, 2005; Hecker et al., 2009a; Gustafsson et al., 2005). More recent approaches also predict scale-free GRNs (Hecker et al., 2009a; Gustafsson and Hörnquist, 2010; Altwasser et al., 2012).

### Differential equation based NI

Systems of ODEs are widely used in physics, chemistry, and biology to describe and model dynamic systems. Yet, ODEs provide an excellent way to mathematically infer GRNs. Here, the expression change of a gene at a time point is modeled as the weighted sum of the expression of all other genes and an external stimuli represents an environmental change. In contrast to steady linear regression models, differential equations model the change of a gene expression, not the gene expression itself.

Differential equation approaches reflect cause-effect relations which can be visualized by directed networks. The environmental change directly leads to fast reacting genes that regulate genes which respond later. Thus, GRNs modeled by ODEs are dynamic with directed and signed (weighted) edges. Similar to linear regression methods, weights are determined by optimization algorithms with the aim to fit measured data. In this NI category, many approaches apply the sparseness criterion and integrate prior knowledge by preferring known interactions during model structure optimization (e.g., Guthke et al., 2005; Greenfield et al., 2013; Zhang et al., 2013). While predicted GRNs are often highly accurate, a drawback is that the approaches are computationally demanding. Thus, they are often applied to a small number of genes which need to be carefully selected (see below).

One approach based on differential equations is NetGenerator (Guthke et al., 2005; Toepfer et al., 2007). The tool applies the sparseness criterion with help of a heuristic search strategy. Furthermore, it softly integrates prior knowledge and has been augmented to predict interactions which are robust against noise in expression data (Linde et al., 2010). NetGenerator was successfully applied to predict GRNs for immune diseases (Guthke et al., 2005), stress adaptation processes of pathogens (Linde et al., 2010, 2012) and rheumatoid arthritis (Kupfer et al., 2014). Since 2013, NetGenerator is able to predict GRNs based on more than one expression data set and more than one stimulus (Weber et al., 2013), which is for example useful for combined drug treatment.

## Dual network inference

**Figure 2:**
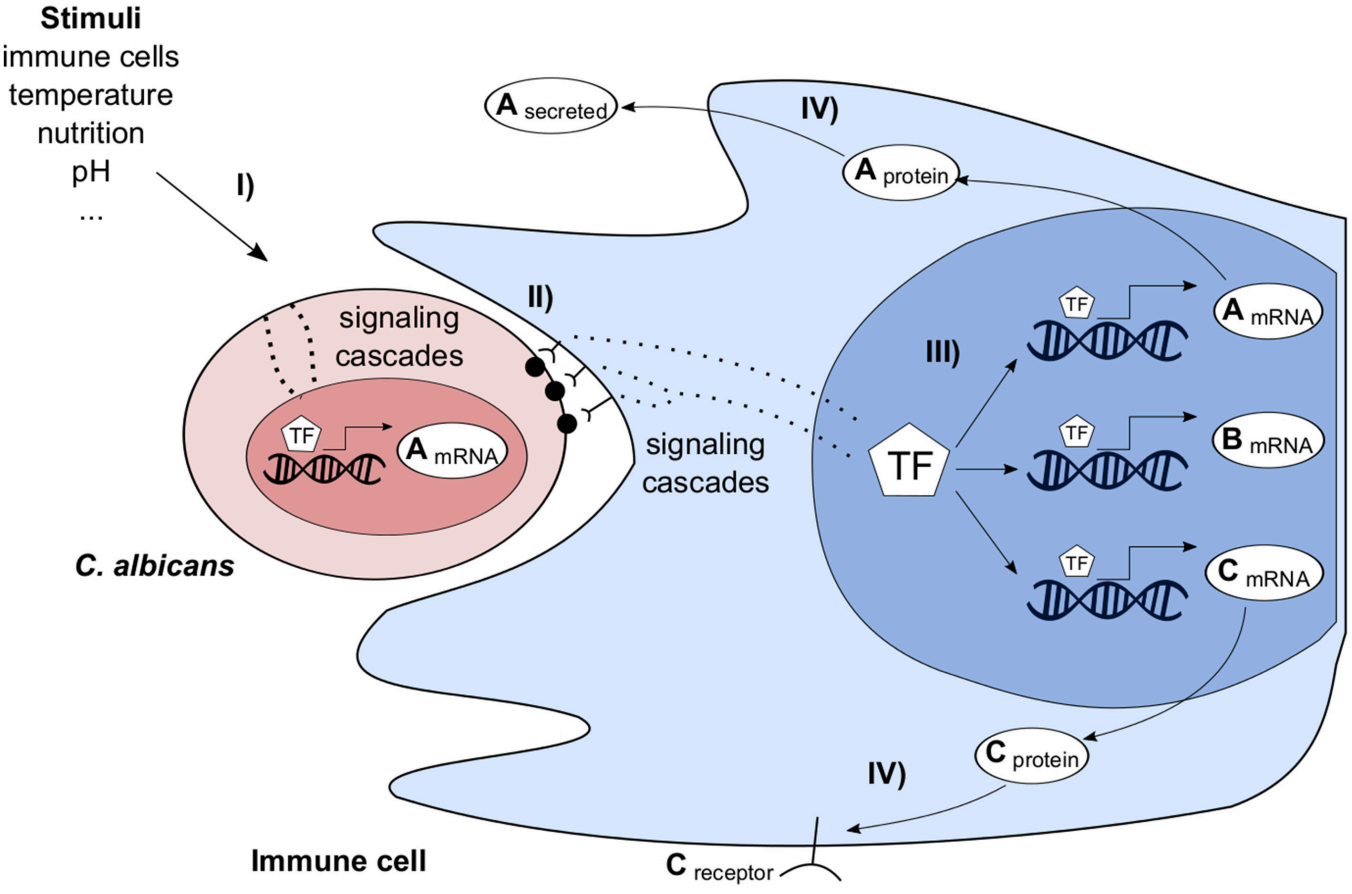
**Simplified overview of ***Candida albicans*** (red) interacting with an immune cell (blue) and its environment**. *C. albicans* is stimulated by environmental factors **(I)** leading to a change of its transcriptome. Immune cells recognize the pathogen, e.g., via pattern recognition receptors **(II)**, transmit the signal through the cell and adapt their transcriptional program **(III)**. In turn, this stimulates *C. albicans*, e.g., by producing cellsurface or extracellular proteins **(IV)**.

Interacting organisms of two or more species form a complex system. Various direct and indirect interactions take place and trigger multiple responses at different scales. In the following, as an example the complex interactions of the pathogenic fungus *C. albicans* and the human immune system are outlined in an abstract and simplified way (Figure 2). *C. albicans* cells can migrate into host tissues where they are exposed to different environmental conditions, such as a change in pH, available nutritions, presence and contact with immune cells. These stimuli are sensed by *C. albicans*, transmitted through the cell and finally the transcriptional program is changed. Similarly, immune and tissue cells sense the presence of *C. albicans* and change their transcriptional program. The transcriptional changes of both species can be measured by dual RNA-seq or microarrays. After the processing of transcribed mRNAs, the host adapts to infection. E.g., membrane bound and soluble receptors for pathogen recognition and cell signaling molecules for cell-cell communication are produced. Furthermore, host defense responses are initiated, such as the generation of reactive oxygen species. In turn, the pathogen again changes its transcriptional program to protect itself against host defense mechanisms. GRNs cannot comprehensively describe all molecular mechanisms of such a complex system of interacting species, but predict essential and also indirect interactions. Tierney et al. (2012) predicted inter-species interactions in a system of murine dendritic cells interacting with *C. albicans*. As outlined before (Figure 2), these predictions are highly indirect but were experimentally validated.

Theoretically, all presented NI methods (see Section 4) can be applied to dual transcriptomics data. Some NI approaches additionally allow for prior knowledge as input. Given these inputs, NI approaches may work with dual or simultaneous gene expression data and finally predict a GRN including genes of both interacting species. Molecular interactions can be predicted between genes of one species (**molecular intra-species interaction**) or between genes of different species (**molecular inter-species interaction**; Figure 1C). NetGenerator (Guthke et al., 2005; Toepfer et al., 2007) is a tool that requires time series data of DEGs in form of a gene expression matrix, which can consist of genes from two interacting organisms. Relevant genes to be incorporated in the inference have to be selected. A maximum number of 20–30 genes is recommended depending on the number of samples. For example, genes can be selected based on their association to enriched Gene Ontology terms or with the help of expert knowledge.

KEY CONCEPT 6 **Molecular intra-species interactions:**

Molecular intra-species interactions are gene regulatory interactions predicted between two genes within one species. These predictions can be direct or indirect.

KEY CONCEPT 7 **Molecular inter-species interactions:**

Molecular inter-species interactions are predictions of a gene from species A interacting with a gene from species B. The predicted inter-species interactions are highly indirect.

To the authors' best knowledge the first practical inter-species application of NI was carried out with NetGenerator based on dual RNA-seq data of *M. musculus* dendritic cells infected with *C. albicans* (Tierney et al., 2012). Guided by this experience, we augmented NetGenerator for typical scenaria of dual transcriptomics data (Schulze et al., 2015) which we will introduce in the following.

A change in gene expression is triggered by one or more stimuli, which NetGenerator integrates through one or more time-dependent functions. Such a function is a user-defined input which represents the environmental change over time. If both species respond immediately, one identical stimulus function for both species might be sufficient. It is also possible, that one species responds faster than the other, which could be translated into two or more stimuli functions. NetGenerator was extended to incorporate multiple stimuli by Weber et al. (2013), while making use of multiple stimuli for dual NI was first discussed in Schulze et al. (2015).

NetGenerator was extended in Schulze et al. (2015) to deal with missing data at intermediate time points of time series. For example, this can occur when no gene expression values can be determined due to insufficient coverage or other technical problems. Furthermore, if transcriptome data from two species are combined retrospectively into a dual transcriptomics data set, time points can differ. Internally, NetGenerator handles this problem by interpolating missing data points. NetGenerator does not accept missing values for the first or last time point. In that case, the user has to provide these values, e.g., by setting them to zero or preceding/succeeding values.

Finally, NetGenerator was extended to consider gene expression variances. Biological variance in gene expression data exists for each experimental setup. However, the complex nature of biotic interactions *in vivo* leads to more variance than in *in vitr*o experiments where under defined conditions only one environmental parameter is changed (e.g., heat shock). For each gene at each time point, a variance is calculated based on replicated measurements. The NI process of NetGenerator is sequential, i.e., it integrates one gene after another. For each gene, an objective function is minimized. In a simplified way, this means that the difference between measured data and simulated time course data should be as small as possible. The extended NetGenerator includes gene expression variances in the objective function. For gene expression values with a large variance, the difference of measured and simulated data is allowed to be larger than for gene expression values with smaller variance.

### Issues and perspectives

Dual transcriptomics paves the way to study the molecular basis of interaction. With the advent of RNA-seq it is now possible to study the transcriptome of non-model species. In this Frontiers Focused Review, we present an overview of dual and simultaneous transcriptomics studies which shows the wide range of possible applications from studying host-pathogen interactions via symbiotic fungal-host interactions to social interactions of insects. Among these studies, there is no example where molecular host-pathogen interactions were solved *omics*-based by both, dual and simultaneous transcriptomics. The majority of theses studies have identified genes and pathways involved in the interaction process. With dual NI, we present an approach which goes beyond identification of DEGs and uses gene expression data to predict molecular inter-species interactions (Figure 1C; Tierney et al., 2012; Schulze et al., 2015). Hypothetic gene regulatory interactions predicted by NI might be indirect. In fact, genes never directly interact. The most direct interaction, which can be predicted is between a transcription factor coding gene and the transcription factors target gene.

Indirect interactions may represent whole pathways or signaling cascades relevant in the interaction between two species. For a long time, indirect interactions have been regarded as a drawback of NI. In fact, the ability to predict indirect interactions is a big advantage, when NI approaches are applied to dual transcriptomics data. Predicted molecular inter-species interactions are by nature indirect but they are extremely interesting as they indicate which gene from species A influences which gene from species B (e.g., *via* one or more hidden molecular mediators, receptors, pathways or transcription factors). In future, dual network inference may also be applied to dual transcriptomics experiments where different organisms of the same species interact with each other (e.g., Vojvodic et al., 2015).

As models are always abstractions from a complex reality they do have a number of simplifications/assumptions. We have already discussed, that correlation of two genes does not mean a causal interaction. Other approaches based on regression or differential equations often assume linearity, which means the concentration of an activated gene is a linear function of its activator. However, biochemical kinetics often contain saturation dynamics (e.g., Michaelis Menten kinetics) or synergistic effects (e.g., Hill kinetics). Some tools also allow for non-linear modeling, e.g., NetGenerator (Guthke et al., 2005). This introduces additional parameters that need to be identified and therewith more prior knowledge or experimental data is needed. On the other hand, the assumption of linearity also holds true for wide ranges of biochemical kinetics.

A general assumption in (dual) transcriptomics is that the measured gene expression changes have an influence on the phenotype. However, proteins catalyze most biochemical reactions and shape the structure of a cell. Dual proteomics may pave the way to directly measure proteins. As the overlap between proteomics and transcriptomics differs depending on the experiment (Haider and Pal, 2013), NI approaches combining both *-omics* approaches are necessary.

Recently, first steps were done toward a systems biology of pathogen-host interactions by combining models of GRNs and signaling networks with models of other levels, in particular by modeling of metabolic and Protein-Protein Interaction (PPI) networks (Durmus et al., 2015; Schleicher et al., 2016). Sometimes, GRN modeling was also supported by prior knowledge retrieved from PPI databases (e.g., Altwasser et al., 2012).

Depending on the number of genes, NI approaches are divided into small scale and large scale NI. Large scale approaches often need a compendium of gene expression data combining different experiments. Thus, predicted interactions are gobal (genome-wide) for the respective interacting species and not specific for a certain condition/treatment. These approaches are useful to identify central genes (hubs) in regulatory networks. Small scale approaches typically focus on certain experimental conditions and are thus useful for dual NI. Even though, statistical (*p*-values for differential expression) and biological (e.g., member of an interesting biological process) methods for gene selection exist, this process is often subjective. Novel NI approaches need to combine advantages of large scale and small scale methods.

When we study the transcriptome of microorganisms, we need to be aware that we measure a mixture of hundreds or billions of cells, that might even be of different cell types. Expression values are a kind of “average” over the expression of all these different cells which assumes one big identical cell population. In fact, there might be sub-populations during the experiment. Moreover, individual cells may follow a very specific strategy during a biotic interaction. Single-cell RNA-seq allows to measure the transcriptome of each individual (Shapiro et al., 2013; Battle et al., 2014) and will change our understanding of molecular inter-species interactions. While single-cell RNA-seq is already possible for higher organisms, methods are being adapted for small RNA amounts of microorganisms. Single-cell RNA-seq of interacting species will help to identify the molecular basis of two interacting cells. Methods for NI based on such data need to take into account variability between different cells of the same species. In comparison to networks from averaged data, networks of a single cell may help to identify genes and interactions which vary between cells and are connected to a specific phenotype. Such an approach is applicable for individualized medicine (Lu et al., 2014).

This Frontiers Focused Review is mainly dedicated to dual RNA-seq data and modeling of two interacting species such as pathogen-host interaction. RNA-seq opens the door for the investigation of multi-species interaction, in particular the interactions between microorganisms and viruses in oral and gut microbiomes (Bikel et al., 2015). Whereas, metagenomics is focused on the relative abundance of the different species (genomes), the emerging RNA-seq-based metatranscriptomics will provide gene expression data of a biome and, thus, the empirical basis for molecular modeling of multi-species population networks. Examples are metatranscriptomics of the human gut (Franzosa et al., 2014) and its application to the current research on inflammatory bowel disease (Valles-Colomer et al., 2016) and the mixed culture of three bacterial species (Giannoukos et al., 2012). In addition, there are examples of metatranscriptome studies in environmental research, such as to analyse the gene expression and dynamics in the environments of the Pacific (Stewart et al., 2011) or the Amazon River (Satinsky et al., 2014).

## Author contributions

Substantial contributions to the conception and design of the work: all. Drafting the work: SS, JS, RG, JL. Revising the work critically for important intellectual content: all. Final approval of the version to be published: all. Agreement to be accountable for all aspects of the work in ensuring that questions related to the accuracy or integrity of any part of the work are appropriately investigated and resolved: all.

## Funding

SS and JL are supported by the Deutsche Forschungsgemeinschaft (DFG) CRC/Transregio 124 Pathogenic fungi and their human host: Networks of interaction subproject B3 (SS) and subproject INF (JL).

### Conflict of interest statement

The authors declare that the research was conducted in the absence of any commercial or financial relationships that could be construed as a potential conflict of interest.
